# Thiol/Disulphide Homeostasis in the Relationship Between Body Condition Score and Oxidative Stress in Periparturient Period Holstein Heifers

**DOI:** 10.1002/vms3.70326

**Published:** 2025-04-04

**Authors:** Tamer Kayar, Guzin Ozkurt, Onur Erzurum, Kubra Er, Beril Buyukgungor, Beyza Nur Gecgel, Muhammet Nureddin Karaburc

**Affiliations:** ^1^ Department of Animal Science, Faculty of Veterinary Medicine Aksaray University Aksaray Türkiye; ^2^ Department of Biochemistry, Faculty of Veterinary Medicine Aksaray University Aksaray Türkiye; ^3^ Karapınar Vocational School Veterinary Department Selcuk University Konya Türkiye

**Keywords:** body condition score, oxidative stress, primiparous heifer, thiol/disulphide homeostasis, transition period

## Abstract

This study aimed to evaluate the effects of body condition scores (BCSs) on oxidative stress and thiol/disulphide homeostasis (TDH) in Holstein pregnant heifers during the transition period. A total of 36 healthy primiparous heifers in the 7th month of pregnancy, all approaching their first calving, were included in the study. The animals were allocated into three equal groups based on their BCS. The BCS measurements were performed 21 days pre‐calving and on the 21st day post‐calving. Pre‐calving and post‐calving serum levels of total thiol (TTL), native thiol (NTL) and disulphide (DSF) were quantified using standard techniques. The study revealed significant differences in BCS, TTL and DSF values among groups both pre‐ and post‐calving (*p* < 0.05), whereas the changes in NTL values were statistically insignificant (*p *> 0.05). Overall, a decrease in BCS and NTL levels was observed, accompanied by an increase in TTL and DSF levels. Correlation analysis within groups indicated low‐level relationships between changes in BCS and TTL, NTL and DSF levels. Consequently, regression analysis did not yield any statistically significant predictive models. The results showed a differential response between the loss of BCS and the oxidative stress during the periparturient period. The increased DSF levels observed during late pregnancy and early lactation indicate a deficiency in antioxidant substances in the animals. Therefore, supplementing the ration with a premix containing antioxidant substances during the transition period may provide significant benefits in terms of maintaining the BCS balance, animal welfare and herd health.

## Graphical abstract

This study aimed to evaluate the effects of body condition scores (BCSs) on oxidative stress and thiol/disulphide homeostasis (TDH) in Holstein pregnant heifers during the transition period. The results showed a differential response between the loss of BCS and the oxidative stress during the periparturient period. The increased DSF levels observed during late pregnancy and early lactation indicate a deficiency in antioxidant substances in the animals.

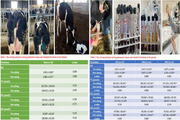

## 1 Introduction

The transition period is one of the most critical stress factors affecting dairy cows. The 3‐week phase preceding calving is referred to as the ‘prepartum period’, whereas the 3‐week phase following calving is termed the ‘postpartum period’. Additionally, the period spanning a few days pre‐calving and post‐calving is known as the ‘periparturient period’ (Arslan and Tufan 2010). Among lactation periods, the transition period is particularly significant due to the metabolic, physiological and nutritional changes it encompasses, including shifts in oxidant/antioxidant balance and nutrient requirements. This period is crucial for the health and productivity of dairy cows, as they are subjected to severe physiological changes and metabolic stress (Goff and Horst 1997; Drackley 1999).

The onset of some metabolic diseases and disorders in high‐yielding dairy cows during the periparturient period is closely associated with the negative energy balance that emerges during this time (Esposito et al. 2014). The primary challenge during this period is the inability of animals to meet the heightened nutrient demands required to sustain peak milk production (Sundrum 2015). Changes in the thiol/disulphide homeostasis (TDH) during the transition period are largely associated with oxidative stress levels. TDH serves as a biomarker indicating the susceptibility of proteins to oxidative modifications and represents a crucial reflection of metabolic stress (Erel and Neşelioğlu 2014). Previous studies have demonstrated that in cows experiencing negative energy balance, the immune system is suppressed, oxidative stress is elevated, and these conditions can adversely affect reproductive performance (Drackley 1999; Abuelo et al. 2013; Esposito et al. 2014).

Understanding the physiological changes occurring during this period and implementing feeding programmes that address the changing nutrient requirements are essential. To achieve this, body condition scoring can be used to develop appropriate nutritional strategies. Body condition score (BCS) provides a quick and effective method to evaluate an animal's energy reserves by assessing fat metabolism and its relationship with energy metabolism (Shah et al. 2019; Ullah et al. 2019). From a nutritional perspective, antioxidant supplementation can be effective in reducing oxidative stress. Specifically, nutrients such as vitamin E, selenium, β‐carotene and methionine play critical roles in regulating TDH (Weiss 1998; Spears and Weiss 2008). Studies have shown that the combination of selenium and vitamin E enhances antioxidant capacity and reduces oxidative damage (LeBlanc et al. 2004).

Recent studies in humans have demonstrated a strong link among oxidative stress, high body mass index and weight loss (Ozata et al. 2002; Higdon and Frei 2003; Keaney et al. 2003). Furthermore, oxidative stress has been implicated in the development of several metabolic disorders (Higdon and Frei 2003; Morrow 2003). Oxidative stress is defined as an imbalance between oxidants and antioxidants at the cellular level (Haydar et al. 2020; Beaupre and Weiss 2021). It arises from the production of harmful free radicals during the digestion of nutrients, which can damage tissues, a process referred to as oxidative stress (Coşkun et al. 2016).

Antioxidants are substances that neutralize free radicals and prevent tissue damage (Çetinkaya 2020). Thiol groups are among the first targets of free radicals. The total thiol (TTL) level is directly proportional to the extent of cellular damage caused by free radicals (Haydar et al. 2020). Certain antioxidants, including ascorbic acid (vitamin C), α‐tocopherol (vitamin E) and ceruloplasmin, as well as trace minerals such as selenium, zinc, iron and copper, act as endogenous antioxidants and are crucial for mitigating oxidative stress (Dimri et al. 2010; Aslankoç et al. 2019). TDH, which reflects the dynamic balance between thiols and disulphides (DSFs), is a reliable marker of oxidative stress. Studies in human medicine increasingly indicate that abnormal thiol/DSF levels contribute to the pathogenesis of various diseases. Although TDH is a relatively new area of research in veterinary medicine, it is gaining attention due to its potential diagnostic and prognostic value (Camkerten et al. 2019; Değirmençay et al. 2021; Terzi et al. 2022; Dursun 2023).

In recent years, Türkiye has imported significant numbers of cattle breeds for breeding and fattening purposes, with pregnant heifers constituting a substantial proportion of these imports (Kayar and İnal 2019, 2022; Kayar and Budak 2023). Although numerous studies have examined oxidative stress in lactating cows, there is limited research that has focused on pre‐ and post‐calving BCS and TDH in pregnant heifers. This study aims to evaluate TDH as an oxidative stress marker in relation to BCS in pregnant heifers during the transition period. The findings are expected to contribute valuable insights to the existing literature and serve as a guide for future research in this field.

## 2 Materials and Methods

The study was conducted at a private dairy farm located in Aksaray Province, Turkey (38°19′11.7″N, 33°54′13.7″E) and received ethical approval from the Aksaray University Animal Experiments Local Ethics Committee (Protocol No. 2024/5‐38).

### 2.1 Determination of BCS and Groups Formation

The study material comprised 36 heifers in the 7th month of pregnancy, all of which were primiparous and confirmed to be healthy based on health screening results. The BCS values of the selected heifers were recorded exactly 21 days pre‐calving and 21 days post‐calving. The BCS values were determined following the method described by Ferguson et al. (1994), utilizing a 5‐point scale with 0.25‐point intervals.

The animals were categorized into three groups based on their BCS:

- Low body condition score (LBCS): ≤3.00 (*n* = 12)
- Medium body condition score (MBCS): 3.25 ≤ BCS ≤ 3.5 (*n* = 12)
- High body condition score (HBCS): ≥3.75 (*n* = 12)

### 2.2 Collection of Blood Samples

Heifers, 21 days pre‐calving according to the insemination records, were selected, and the BCS of each animal was determined. Blood samples were collected for each animal on the 21st day pre‐calving and on the 21st day post‐calving. Blood samples were taken from the jugular vein into 9 mL serum tubes according to standard technique. The samples were transported to the laboratory while adhering to the cold chain protocol. The blood samples in the serum tubes were centrifuged at 3000 rpm for 10 min. The resulting sera were then transferred to duplicate Eppendorf tubes and stored at −20°C until the date of analysis.

### 2.3 Determination of TDH

Serum TDH was measured colourimetrically, following the method described by Erel and Neşelioğlu (2014). In the first step, only dynamic and reducible DSF bonds were fully reduced to free functional thiol groups, whereas static and structural DSF bonds remained unaffected. In the second step, any remaining unused reducing agent, NaBH_4_ (sodium borohydride), was completely consumed. In the final step, all thiol groups, including both native and reduced thiol groups, were reacted with DTNB [5,5′‐dithiobis (2‐nitrobenzoic acid)] using Ellmann's reagent. The number of DSF bonds was calculated as half of the difference between the TDH and native thiol (NTL) levels.

### 2.4 Statistical Analysis

One‐way analysis of variance (ANOVA) was conducted to compare thiol/DSF levels across different BCS groups. Additionally, paired *t*‐tests were used to assess differences in BCS and thiol parameters between pre‐ and post‐calving measurements within each group. Pearson correlation coefficients and simple linear regression analyses were performed to examine the relationships among the variables. The statistical significance threshold was set at 5%, and all statistical analyses were carried out using SPSS v.21 software. The correlations between the BCS and thiol values measured exactly 21 days pre‐calving and 21 days post‐calving were calculated using the regression equation formula as follows:

Y=α+βX

where *Y* is the dependent variable, *α* is the constant, *β* is the intercept, and *X* is the BCS1, BCS2.

The criteria for interpreting the correlation between variables *X* and *Y* were as follows (Laras et al. 2024):

- 0.00–0.199: very low correlation
- 0.20–0.399: low correlation
- 0.40–0.599: medium correlation
- 0.60–0.799: high correlation
- 0.80–1.000: very high correlation

## 3 Results

The differences among the groups in terms of mean BCS at 21 days pre‐calving and 21 days post‐calving were found to be statistically significant (*p* < 0.05). Decreases in BCS were observed in all animals post‐calving. However, these changes in BCS did not lead to significant differences in the levels of TTL, NTL and DSF in the serum samples obtained from the same animals on the respective days (*p* > 0.05). Upon examining the overall averages of the parameters, a decrease in NTL levels and an increase in TTL and DSF levels were noted (Table 1).

**TABLE 1:** Pre‐ and post‐calving parameters means and standard deviations of all animals.

Parameters	Mean (*n* = 36)	*p* value
**BCS**		
Pre‐calving	3.403 ± 0.420^a^	0.000
Post‐calving	2.888 ± 0.371^b^	0.000
**TTL (µmol/L)**		
Pre‐calving	267.555 ± 109.454	0.155
Post‐calving	509.972 ± 70.191	0.055
**NTL (µmol/L)**		
Pre‐calving	159.361 ± 61.649	0.782
Post‐calving	148.167 ± 56.535	0.771
**DSF (µmol/L)**		
Pre‐calving	54.097 ± 44.297	0.135
Post‐calving	180.903 ± 45.256	0.312

*Note*: Different superscript letters in the same column indicate statistically significant differences among groups (p < 0.05).

Abbreviations: BCS, body condition score; DSF, disulphide; NTL, native thiol; TTL, total thiol.

When comparing the parameters within each group, significant differences were observed in BCS, TTL and DSF values pre‐ and post‐calving (*p* < 0.05), whereas no statistically significant differences were found in NTL values (*p* > 0.05). Overall, BCS and NTL levels tended to decrease, whereas TTL and DSF levels exhibited an increase (Table 2).

**TABLE 2:** Pre‐ and post‐calving parameters means and standard deviations in the groups.

Parameters	LBCS (*n* = 12)	MBCS (*n* = 12)	HBCS (*n* = 12)
**BCS**			
Pre‐calving	2.917 ± 0.123^a^	3.417 ± 0.123^a^	3.875 ± 0.169^a^
Post‐calving	2.542 ± 0.144^b^	2.875 ± 0.199^b^	3.250 ± 0.320^b^
***p* value**	0.000	0.000	0.000
**TTL (µmol/L)**			
Pre‐calving	245.167 ± 98.265^b^	240.167 ± 82.216^b^	317.333 ± 132.797^b^
Post‐calving	514.250 ± 49.424^a^	541.667 ± 42.358^a^	474.000 ± 94.402^a^
***p* value**	0.000	0.000	0.007
**NTL (µmol/L)**			
Pre‐calving	150.167 ± 45.148	159.667 ± 79.423	168.250 ± 59.731
Post‐calving	155.750 ± 57.479	149.833 ± 56.472	138.917 ± 59.310
***p* value**	0.747	0.650	0.139
**DSF (µmol/L)**			
Pre‐calving	47.500 ± 48.016^b^	40.250 ± 27.438^b^	74.542 ± 49.723^b^
Post‐calving	179.250 ± 45.907^a^	195.917 ± 39.617^a^	167.542 ± 48.949^a^
***p* value**	0.000	0.000	0.002

*Note*: Different superscript letters in the same column indicate statistically significant differences among groups (p < 0.05).

Abbreviations: HBCS, high body condition scores group; LBCS, low body condition scores group; MBCS, medium body condition scores group.

Correlations between BCS and thiol parameters in the different groups are presented in Table 3. In the LBCS group, the correlations between pre‐calving BCS and TTL (*r* = 0.396) and DSF (*r* = 0.354) were low. A very low correlation was observed between BCS and NTL (*r* = 0.109). These correlation values were even lower post‐calving. A strong positive correlation was found between pre‐calving and post‐calving TTL (*r* = 0.892) and DSF (*r* = 0.834). A negative correlation between DSF and NTL was observed, which became more pronounced post‐calving (*r* = −0.880).

**TABLE 3:** Correlations of dependent variables with pre‐calving and post‐calving body condition scores in the groups.

		BCS1	TTL1	NTL1	DSF1	BCS2	TTL2	NTL2	DSF2
	**BCS1**	1							
	**TTL1 (µmol/L)**	0.396	1						
	**NTL1 (µmol/L)**	0.109	0.279	1					
**LBCS**	**DSF1 (µmol/L)**	0.354	0.892^**^	−0.185	1				
	**BCS2**	0.533	0.238	0.156	0.171	1			
	**TTL2 (µmol/L)**	0.004	0.170	0.052	0.149	0.161	1		
	**NTL2 (µmol/L)**	−0.074	−0.031	0.369	−0.206	−0.155	−0.472	1	
	**DSF2 (µmol/L)**	0.048	0.111	−0.203	0.209	0.184	0.834^**^	−0.880^**^	1

		BCS1	TTL1	NTL1	DSF1	BCS2	TTL2	NTL2	DSF2
	**BCS1**	1							
	**TTL1 (µmol/L)**	0.197	1						
	**NTL1 (µmol/L)**	0.311	0.770^**^	1					
**MBCS**	**DSF1 (µmol/L)**	−0.155	0.384	−0.294	1				
	**BCS2**	0.231	0.382	0.480	0.008	1			
	**TTL2 (µmol/L)**	0.151	0.065	−0.063	0.188	−0.121	1		
	**NTL2 (µmol/L)**	0.399	0.021	0.465	−.642^*^	0.389	−0.271	1	
	**DSF2 (µmol/L)**	−0.204	0.020	−0.365	0.558	−0.342	0.727^**^	−.857^**^	1

		BCS1	TTL1	NTL1	DSF1	BCS2	TTL2	NTL2	DSF2
	**BCS1**	1							
	**TTL1 (µmol/L)**	−0.516	1						
	**NTL1 (µmol/L)**	−0.313	0.713^**^	1					
**HBCS**	**DSF1 (µmol/L)**	−0.501	0.907^**^	0.352	1				
	**BCS2**	0.843^**^	−0.252	−0.092	−0.282	1			
	**TTL2 (µmol/L)**	0.114	−0.021	0.185	−0.139	−0.068	1		
	**NTL2 (µmol/L)**	−0.006	0.433	0.427	0.322	−0.007	0.254	1	
	**DSF2 (µmol/L)**	0.114	−0.283	−0.080	−0.329	−0.061	0.810^**^	−0.361	1

*Correlation is significant *p* < 0.05 level

**Correlation is very significant *p* < 0.01 level.

Abbreviations: BCS1, body condition score 1; BCS2, body condition score 2; DSF1, disulphide 1; DSF2, disulphide 2; HBCS, high body condition scores group; LBCS, low body condition scores group; MBCS, medium body condition scores group; NTL, native thiol; NTL2, native thiol 2; TTL1, total thiol 1; TTL2, total thiol 2.

In the MBCS group, the correlations of BCS with TTL and DSF in the pre‐ and post‐calving periods were very low (*r* < 0.20), whereas its correlation with NTL was moderate (*r* < 0.40). The correlation between pre‐calving BCS and DSF was negative (*r* = −0.155). A high positive correlation was found between pre‐calving TTL and NTL (*r* = 0.770). As in the LBCS group, a strong positive correlation between post‐calving TTL and DSF (*r* = 0.727) and a high negative correlation between DSF and NTL (*r* = ‐0.857) were observed in this group.

There were similar findings in the HBCS group. However, the relationships between pre‐calving BCS and TTL, NTL and DSF were negative. The correlations were moderate for TTL and DSF (*r* ← 0.599) and low for NTL (*r* < 0.399). A very high positive correlation between pre‐calving and post‐calving DSF and TTL was found (*r* = 0.907; *r* = 0.810). A negative correlation between DSF and NTL post‐calving was observed, but this correlation was low (*r* = −0.361).

In general, pre‐calving and post‐calving changes in BCS exhibited low correlations with TTL, NTL and DSF in all groups. For this reason, a statistically significant regression equation could not be obtained from the simple linear regression analysis (*p* > 0.05) (Tables 4 and 5).

**TABLE 4:** Regression analyses of pre‐calving body condition scores with dependent variables in groups.

	Variable	Constat	*β*	*R*	*R* ^2^	*Y*	*p* value
	**TTL1**	−676.50	316.00	0.40	0.16	*y* = −676.5 + 316*x*	0.34
**LBCS**	**NTL1**	33.50	40.00	0.11	0.12	*y* = 33.5 + 40*x*	0.92
	**DSF1**	−355.00	138.00	0.35	0.13	*y* = 355.5 + 138*x*	0.32
	**TTL1**	−209.13	131.50	0.20	0.40	*y* = −209.1 + 131.5*x*	0.77
**MBCS**	**NTL1**	−525.38	200.50	0.31	0.10	*y* = −525.4 + 200.5*x*	0.45
	**DSF1**	158.13	−34.50	−0.16	0.02	*y* = 158.1 − 34.5*x*	0.52
	**TTL1**	1892.13	−406.40	0.52	0.27	*y* = 1892.1 − 406.4*x*	0.55
**HBCS**	**NTL1**	597.60	−110.80	0.31	0.10	*y* = 597.6 − 110.8*x*	0.18
	**DSF1**	647.27	−147.80	0.50	0.25	*y* = 647.3 − 147.8*x*	0.07

*Note*: Statistically significant when *p* values are <0.05.

Abbreviations: BCS1, body condition score 1; DSF1, disulphide 1; HBCS, high body condition scores group; LBCS, low body condition scores group; MBCS, medium body condition scores group; NTL1, native thiol 1; TTL1, total thiol 1.

**TABLE 5:** Regression analyses of post‐calving body condition scores with dependent variables in groups.

	Variable	Constant	*β*	*R*	*R* ^2^	*Y*	*p* value
	**TTL2**	374.23	55.09	0.16	0.03	*y* = 374.2 + 55.1*x*	0.20
**LBCS**	**NTL2**	312.41	−61.64	−0.15	0.02	*y* = 312.4 − 61.6*x*	0.35
	**DSF2**	30.91	58.36	0.18	0.03	*y* = 30.9 + 58.4*x*	0.90
	**TTL2**	615.60	−25.71	−0.12	0.02	*y* = −615.6 − 25.7*x*	0.11
**MBCS**	**NTL2**	−167.24	110.29	0.39	0.15	*y* = −167.2 + 110.3*x*	0.50
	**DSF2**	391.42	−68.00	−0.34	0.12	*y* = 391.4 − 68*x*	0.14
	**TTL2**	539.00	−20.00	−0.07	0.01	*y* = 539 − 20*x*	0.11
**HBCS**	**NTL2**	143.25	−1.33	−0.01	0.00	*y* = 143.3 − 1.3*x*	0.47
	**DSF2**	197.88	−9.33	−0.06	0.00	*y* = 197.9 − 9.3*x*	0.24

*Note*: Statistically significant when *p* values are <0.05.

Abbreviations: BCS2, body condition score 2; DSF2, disulphide 2; HBCS, high body condition scores group; LBCS, low body condition scores group; MBCS, medium body condition scores group; NTL2, native thiol 2; TTL2, total thiol 2.

## 4 Discussion

The BCS determines the amount of fat or energy reserves of all animals in the herd at different periods and at certain intervals. BCS in dairy cattle is an important management tool to reduce the incidence of metabolic and other peripartum diseases. During the last 21 days pre‐calving and at the time of calving, cows should ideally have a BCS of 3.0–3.5 (Şahin 2024). This is necessary for cows to maintain energy reserves and minimise energy imbalances during and post‐calving. High body condition at calving (BCS > 4.0), often resulting in reduced feed intake and increased peripartum problems. Low body condition at calving (BCS < 3.0) usually results in a lower milk yield peak and lower milk yield throughout the lactation. The first few weeks post‐calving is a critical period for cows. Depending on the amount of energy required for the onset of lactation and milk production, a decrease in BCS can be observed. BCS should be between 2.5 and 3.0 during this period (Şahin 2024).

In the study, the differences among the groups in terms of BCS averages were statistically significant (*p* < 0.05) (Table 2). The change in mean BCS for all groups pre‐ and post‐calving was approximately 0.52 points (Table 1), and BCS values were close to the ideal range as previously suggested. Specifically, the LBCS, MBCS and HBCS groups experienced decreases of 0.38, 0.54 and 0.63 points in BCS, respectively. Consistent with this study, many researchers have reported that cows with HBCS experience greater weight and condition loss during the transition period compared to cows with LBCS (Bernabucci et al. 2005; Jamali Emam Gheise et al. 2017; El‐Sharawy et al. 2019). Jamali Emam Gheise et al. (2017) also reported that cows with HBCS exhibited lower antioxidant activity; however, they found that the total antioxidant capacity (TAC) was not affected by pre‐calving BCS. In a study by Dobbelaar et al. (2010) investigating the effects of vitamin E supplementation on BCS and oxidative stress in heifers, it was found that serum d‐ROM (determinable free radicals) levels tended to be higher in heifers with HBCS. However, they also concluded that an HBCS and its loss, by themselves, do not directly indicate increased oxidative stress.

TDH is used as an important marker to assess oxidative stress states and antioxidant capacity. TDH plays a key role in antioxidant defence. Therefore, in recent years, TDH has become important in human medicine, and there are many studies on this subject (Ozler et al. 2015; Ateş et al. 2016; Üstüner 2018; Öktem et al. 2021). Related to this research, there are also studies showing the relationship between obesity and oxidative stress in humans. Some researchers have suggested that obesity and high body mass index are associated with oxidative stress, which may be linked to obesity‐related diseases and insulin resistance (Keaney et al. 2003; Higdon and Frei 2003). Obesity, high body mass index and excess weight loss have been associated with increased systemic oxidative stress (Ozata et al. 2002; Chan et al. 2002; Morrow 2003).

Oxidative stress in veterinary medicine, particularly in ruminants, is a relatively recent area of research. However, the interest in TDH and the number of research studies on these topics are increasing day by day (Terzi et al. 2022; Dursun 2023). Oxidative stress is one of the primary causes of immune and inflammatory disorders, increasing the susceptibility of dairy cows to diseases, particularly during the transition period. Dairy cows are more prone to metabolic issues and oxidative stress during pregnancy and lactation (Ullah et al. 2020). Negative energy balance in early lactation is a common physiological cause of oxidative stress and health problems in dairy animals (Elsayed et al. 2019). Proteins and lipids undergo oxidation as a result of oxidative stress in animals. Antioxidant enzymes help mitigate lipid peroxidation and the associated damage by neutralizing free radicals (Sordillo and Aitken 2009). Thiol groups (SH) play an important role in intracellular and extracellular antioxidant defence. DSF bonds (S–S) are formed as a result of oxidation of thiols, and the amount of DSF bonds increases as the oxidative stress in the cell increases. TTL levels are expected to be low in individuals with low oxidative stress (Gumusyayla et al. 2016). Dursun (2023), in his study investigating the relationship between TTL value and fertility in Hair Goats, stated that the rate of multiple births and fertility increased in Hair Goats with low TTL value in the mating season, whereas pregnancy did not occur in those with high TTL value. Bernabucci et al. (2002) reported that season did not change plasma oxidative markers; however, they detected higher SH in erythrocytes of cows exposed to heat more in summer, which may indicate oxidative stress. Bernabucci et al. (2005) reported an increase in plasma thiol groups pre‐calving and a decrease in plasma thiol groups post‐calving in another study investigating the relationship between BCS and oxidative status and metabolic status during the transition period in dairy cows. On the contrary, in this study, an increase in TTL was observed post‐calving in all three groups. Post‐calving DSF also increased significantly. There was a decrease in NTL rather than an increase. TTL has been defined as the sum of NTL and DSF (Erel and Erdoğan 2020). Consequently, the high TTL can be explained by the increase in DSF. This may also be an indicator of the presence of oxidative stress in each of the study groups. Post‐calving, despite the decrease in BCS, the increase in TTL and DSF levels may result from metabolic adaptation mechanisms. The elevated levels of free radicals in the body can trigger the oxidation of thiol groups, leading to the formation of DSF bonds. This condition may be associated with post‐calving energy imbalance and coincides particularly with periods of increased oxidative stress (Bernabucci et al. 2005).

The increased energy demand and reduced dry matter intake following parturition lead to a negative energy balance. Consequently, enhanced fat mobilization leads to increased levels of non‐esterified fatty acids (NEFA) and *β*‐hydroxybutyric acid (BHB) in the blood. The metabolism of NEFA through β‐oxidation can contribute to the generation of reactive oxygen species (ROS), potentially leading to oxidative stress (Ospina et al. 2010; Tsuchiya et al. 2020). This phenomenon suggests that oxidative stress might be further induced, activating antioxidant defence mechanisms. Evaluating thiol and lipid peroxidation parameters together could provide a better understanding of the relationship between BCS and oxidative stress. If the observed increase in TTL and DSF levels is an adaptive response, it may indicate an upregulated antioxidant defence system in the animal. However, it should not be overlooked that excessive oxidative stress may compromise immune function and disrupt energy metabolism, thereby predisposing cows to an increased risk of postpartum disorders such as mastitis, metritis, infertility, ketosis and fatty liver (Contreras and Sordillo 2011; Turk et al. 2012).

Balkan et al. (2024), in their study on Simmental and Montofon breed cows, observed higher TTL, NTL and DSF levels post‐calving in both breeds, consistent with the present study. They reported that the total antioxidant status (TAS) was lower, and the oxidative stress index (OSI) was higher post‐calving, attributing this to a disruption in redox balance in dairy cows post‐calving. Omidi et al. (2017), in their study on plasma TAS, TTL and malondialdehyde (MDA) levels in dairy cows during different stages of lactation, noted that TAS is a suitable indicator for measuring the cumulative effects of antioxidants.

In the current study, pre‐calving and post‐calving BCS changes were found to be weakly correlated with TTL, NTL and DSF in all groups. Consequently, no statistically significant regression equations were derived from the simple linear regression analysis (*p* > 0.05). However, the formulas obtained in this study are presented in Tables 4 and 5, as they were not provided in previous studies investigating the relationship between BCS and thiol levels. The high positive correlation between pre‐calving and post‐calving DSF and TTL was noteworthy in all three groups (*r* > 0.799). A negative correlation between DSF and NTL was also observed, with the negative correlation becoming stronger post‐calving in all groups except for the HBCS group (r = ‐0.80). Thiols are highly sensitive to free radicals due to their sulfhydryl (SH) groups (Erel and Erdoğan 2020). The findings of this study suggest that the increase in TTL levels during late pregnancy and early lactation may be associated with the increase in DSF levels. The elevated free radical levels may enhance the formation of DSF bonds in enzymes and low‐molecular‐weight thiols containing SH groups, indicating the presence of oxidative stress in animals. Bernabucci et al. (2002) examined the association between oxidative status and metabolic status in dairy cows during the transition period. Their findings indicated that, in contrast to the results of the present study, plasma and erythrocyte SH groups and superoxide dismutase (SOD) levels decreased, whereas reactive oxygen metabolite levels increased post‐calving. Invernizzi et al. (2019) reported a positive correlation between oxidative stress indices (ROS, serum antioxidant capacity) and parameters related to negative energy balance (blood free fatty acids (FFA) and *β*‐ (BHB)), especially during the dry period and post‐calving. Similarly, El‐Sharawy et al. (2019) reported that changes in BCS were associated with markers such as BHB (*r* = ‐0.416, *p* < 0.01), MDA (*r* = 0.445, *p* < 0.01) and cholesterol (*r* = 0.342, *p* < 0.01), and they emphasized that BCS loss could have significant consequences on blood metabolism and oxidative status.

## 5 Conclusion

This study aimed to investigate the relationship between BCS and serum TTL, NTL and DSF levels in pregnant Holstein heifers during the transition period. Monitoring changes in BCS is important to keep cows healthy and for milk production. In this respect, it may be advisable to adjust feeding strategies to ensure that cows have sufficient energy reserves pre‐calving and maintain their energy balance post‐calving. The results of the study showed that there may be different interactions between BCS losses and oxidative stress in the periparturient (pre‐calving and post‐calving) period. It was observed that the DSF level increased significantly in the late pregnancy and early lactation period, and an increase in TTL was observed accordingly. All these findings may indicate the presence of oxidative stress and insufficiency of antioxidant substances in animals during this period. Therefore, it has been concluded that supplementing the diet with antioxidant vitamins (E, A and C) and minerals that act as cofactors for antioxidant enzymes (Se, Cu, Zn and Mn) during the transition period may play a crucial role in maintaining BCS balance and managing negative energy balance. Evaluation of oxidative stress markers during this period can be used as a management tool for herd health and animal welfare. However, it may be more appropriate to evaluate TDH together with other lipid biomarkers such as MDA, paraoxonase 1 (PON‐1), triglycerides (TG), high‐density lipoprotein (HDL) and low‐density lipoprotein (LDL) to reveal the relationship between oxidative stress and metabolic status in transitional dairy cows. Therefore, more studies are needed to investigate the relationships between thiol/DSF and oxidant‐antioxidant status.

## Author Contributions

Tamer Kayar and Kubra Er designed the study. Tamer Kayar, Kubra Er, Beril Buyukgungor, Beyza Nur Gecgel and Muhammed Nureddin Karaburc identified the animals for inclusion in the study and classified them into groups based on BCS. Tamer Kayar and **Guzin Ozkurt** collected blood samples, obtained serum, conducted chemical analyses and interpreted the results. Statistical analysis was carried out by Tamer Kayar and Onur Erzurum. All authors contributed to the interpretation of the results and the drafting of the manuscript. The final manuscript was written by Tamer Kayar and approved by all authors.

## Ethics Statement

Present study was carried out in a private dairy farm and was approved by the Aksaray University Animal Experiments Local Ethics Committee (Protocol No. 2024/5‐38).

## Consent

Consent from all animal owners was obtained before conducting the study. All authors have read and approved the final version of the manuscript for submission. They confirm that the article is the authors’ original work, has not been previously published and is not under consideration elsewhere.

## Conflicts of Interest

The authors declare no conflicts of interest.

### Peer Review

The peer review history for this article is available at https://publons.com/publon/10.1002/vms3.70326

## Data Availability

The data supporting the findings of this study are available from the corresponding author upon request.
